# BRASH (Bradycardia, Renal Failure, Atrioventricular Nodal Blockade, Shock, and Hyperkalemia) Syndrome: A Frequently Underrecognized Condition Often Confused With Simple Hyperkalemia

**DOI:** 10.7759/cureus.68106

**Published:** 2024-08-29

**Authors:** Karandeep Singh, Kunal N Patel

**Affiliations:** 1 Internal Medicine, Government Medical College, Amritsar, Amritsar, IND; 2 Cardiology, University of Kansas Medical Center, Kansas, USA

**Keywords:** acute renal failure and hemodialysis in icu, cardiology, av nodal blockers, bradycardia, hyperkalemia, brash syndrome

## Abstract

BRASH syndrome, defined by bradycardia, renal failure, atrioventricular nodal blockade, shock, and hyperkalemia, is a relatively new and often underrecognized condition. In this article, we present a case of an elderly female who developed an episode of syncope. She was found to have refractory shock and bradycardia in the emergency department. Laboratory results and other findings led to the diagnosis of a BRASH syndrome, which was refractory to medical therapy alone, requiring transvenous pacing, hemodialysis, and vasopressor support.

## Introduction

BRASH syndrome, defined by bradycardia, renal failure, atrioventricular nodal blockade, shock, and hyperkalemia, is a relatively new entity and often underrecognized condition [1]. Its most common presentation ranges from asymptomatic to bradycardia [2]. It is usually misdiagnosed and treated as conventional bradycardia, which will not respond to standard drugs for bradycardia such as atropine, thereby potentially leading to cardiovascular collapse and ultimately can lead to shock and multiorgan failure [3], which can cause mortality [4]. It occurs commonly in the older population with multiple comorbidities, especially renal and cardiac comorbidities. Almost all the patients who develop BRASH syndrome are on AV node blockers (AVNB) [5], with an average age of presentation around 69 years [6]. The synergistic effect of AVNB and renal insufficiency leads to hyperkalemia and accumulation of AVNB, eventually resulting in bradycardia and hypotension, which is a basic pathophysiology of BRASH syndrome. Effective management focuses on addressing the underlying cause, which is withholding AVNB agents and providing supportive care. This is done by treating hyperkalemia, providing hemodynamic support, improving renal function, and performing fluid resuscitation for hypovolemic patients. This case includes a hospital visit for BRASH syndrome, for which the patient was managed with varying approaches.

## Case presentation

**Figure 1:**
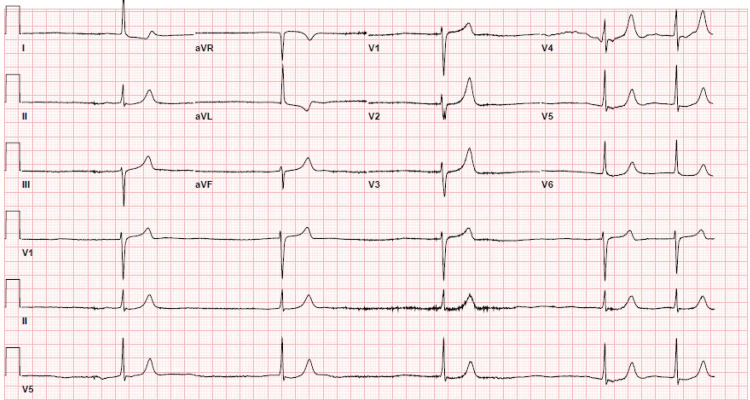
Admission EKG showing atrial fibrillation with a ventricular rate of 31 bpm and peaked T-waves EKG: Electrocardiogram.

An 82-year-old female with a past medical history of hypertension, diabetes mellitus, and paroxysmal atrial fibrillation was found unresponsive at a nursing home. She was brought to the hospital due to loss of consciousness. On admission, her vitals were unstable with blood pressure (BP) of 99/34 mmHg and symptomatic bradycardia with a heart rate (HR) of 33 beats per minute (bpm). She was found to be in a refractory shock. Her temperature was 98.30 F with a respiratory rate (RR) of 24/min. A physical examination showed a moderately, nourished patient with apical HR of 30-35 bpm. Bilateral lungs were clear on auscultation. Her abdomen was soft and non-tender. The other system examinations were normal. She was taking 100 mg metoprolol and 120 mg verapamil daily for her atrial fibrillation. There was no recent dose adjustment for her AVNB drugs, and she was on her stable dose for the past two years. Her blood workup revealed serum creatinine of 1.98 mg/dl (baseline 0.9), blood urea nitrogen of 45 mg/dL, potassium of 6.8 mEq/dL, TSH of 2.9 µIU/ml, and troponin I of 0.2 ng/ml (Table 1). Her electrocardiogram (EKG) showed an atrial fibrillation rhythm with a ventricular rate of 31 bpm and peaked T-waves (Figure 1).

**Table 1:** Laboratory investigations during admission

Parameters	Patient values	Reference range
Potassium (K^+^)	6.8 mEq/dL	3.5-5.0 mEq/L
Creatinine	1.98 mg/dl	0.6-1.2 mg/dL
Blood urea nitrogen (BUN)	45 mg/dL	7-18 mg/dL
Thyroid-stimulating hormone (TSH)	2.9 µIU/ml	0.4-4.0 μIU/m
Troponin I	0.2 ng/ml	<0.04 ng/dL

Chest radiography showed no evidence of active disease, and she had no signs of infection. The echocardiogram showed normal left ventricular systolic function. In the setting of hyperkalemia, bradycardia, and acute kidney injury along with the use of AVNB agents, a differential diagnosis of BRASH syndrome was made. Other differentials were isolated hyperkalemia, isolated AVNB agent toxicity, and adrenal insufficiency. She was intubated for airway protection. Owing to her symptomatic bradycardia and refractory shock, AVNB agents were immediately withheld. For her hyperkalemia along with EKG changes and clinical status, potassium-lowering agents such as insulin, dextrose, and sodium polystyrene sulfonate were given. Her refractory hypotension necessitated the use of intravenous (IV) vasoactive drugs. Thus, IV norepinephrine infusion was started to maintain the mean arterial pressure. Persistent bradycardia with hemodynamic instability prompted us to start transcutaneous pacing, which was futile; hence, a temporary transvenous pacemaker was placed. Temporary hemodialysis was started for worsening renal failure and refractory hyperkalemia. Over the next 24 hours, her clinical status improved, and she was weaned off vasopressor support and mechanical ventilation. Hemodialysis and temporary pacing were discontinued over the next few days. Renal function improved and HR stabilized at 70 bpm. Her verapamil was discontinued, and her metoprolol dose was reduced to 50 mg daily upon discharge. In the follow-up labs, her kidney function and potassium level remained within normal limits.

## Discussion

The BRASH syndrome manifests in patients on AVNB agents who develop acute kidney failure. It is a relatively new entity, and very little is known about its epidemiology [1]. The BRASH syndrome is more frequently seen in older adults with a history of heart and kidney issues. It can be triggered by factors such as dehydration, higher doses of medications that may lead to hyperkalemia and kidney damage, and low BP. A thorough clinical history, with a particular focus on pharmacological treatments, significant bradycardia, and hyperkalemia, is essential in diagnosing a patient with BRASH syndrome [2].

The diagnosis of BRASH syndrome is primarily based on clinical manifestations, EKG findings, and metabolic assessment, after ruling out other potential causes [7]. Patients typically take prescribed doses of AVNB agents; however, renal failure from any cause can lead to the accumulation of these renally cleared drugs. This, along with hyperkalemia, ultimately results in refractory bradycardia.

In our case, the patient likely overdosed on her AVNB agents, metoprolol and verapamil, which she was taking for atrial fibrillation. This overdose caused her HR to drop to 33 bpm, leading to acute kidney injury (creatinine 1.68 mg/dL) and subsequent hyperkalemia (potassium 6.8 mmol/L). Moreover, as metoprolol and verapamil are renally cleared drugs, acute kidney injury resulting from bradycardia led to elevated concentrations of these medications, further exacerbating the bradycardia. These events culminated in the development of BRASH syndrome.

It is crucial to promptly identify and treat this bradycardia, as it does not respond to standard bradycardia medications like atropine. One differential diagnosis to consider is hyperkalemia, which can indeed cause bradycardia, but it typically requires a significantly elevated serum potassium level to do so [8]. Based on laboratory values and the clinical picture, adrenal insufficiency can present with a similar profile to BRASH syndrome. However, thorough history-taking and the presence of hyponatremia along with low cortisol levels can help in making the correct diagnosis [9].

A detailed assessment of the patient's medical history, including any underlying autoimmune conditions or prolonged steroid use, combined with these specific lab findings, can guide clinicians in distinguishing between these two conditions. Consequently, we have to resort to temporary transcutaneous or transvenous pacing, which was the approach taken for our patient as well [10,11]. Reduced cardiac output due to bradycardia will further decrease renal perfusion, which aggravates hyperkalemia and hence puts the patient into a vicious cycle. If this cycle is not addressed on time, it can lead to multiorgan failure and shock [11].

Hyperkalemia should be treated promptly, and patients should receive hemodynamic support with IV fluid to improve renal perfusion. When hyperkalemia is refractory to standard treatments and renal function worsens, dialysis becomes a viable and effective option for managing the condition, which was done for our patient [12]. The cornerstone of management is to stop AVNB and address the precipitating cause. Most mild BRASH syndrome cases respond favorably to basic medical intervention. Early detection of this disease improves prognosis and reduces the need for invasive procedures [10]. Although most patients respond well to medical management, 20% of patients require renal replacement therapy and 33% require transvenous or transcutaneous pacing [6].

## Conclusions

In conclusion, this case highlights the importance of recognizing BRASH syndrome promptly in patients presenting with resistant bradycardia, hyperkalemia, and renal failure. Early diagnosis is crucial for initiating appropriate management, which can significantly impact patient outcomes. This underscores the need for increased awareness among healthcare providers. Understanding the pathophysiology and early intervention strategies can help improve prognosis and reduce morbidity associated with this syndrome.
